# Impact of Personal Protective Equipment on the First-Pass Success of Endotracheal Intubation in the ED: A Propensity-Score-Matching Analysis

**DOI:** 10.3390/jcm10051060

**Published:** 2021-03-04

**Authors:** Jeonghyun Choi, Tae Gun Shin, Jong Eun Park, Gun Tak Lee, Young Min Kim, Soo Ah Lee, Seonwoo Kim, Na Young Hwang, Sung Yeon Hwang

**Affiliations:** 1 Samsung Medical Center, Department of Emergency Medicine, Sungkyunkwan University School of Medicine, Seoul 06351, Korea; jeonghyun1.choi@samsung.com (J.C.); taegunshin@skku.edu (T.G.S.); jebbfirst@gmail.com (J.E.P.); zenky07@naver.com (G.T.L.); ym-em.kim@samsung.com (Y.M.K.); lcl09011@hanmail.net (S.A.L.); 2 Department of Emergency Medicine, College of Medicine, Kangwon National University, Chuncheon 24289, Korea; 3 Statistics and Data Center, Research Institute for Future Medicine, Samsung Medical Center, Sungkyunkwan University School of Medicine, Seoul 06351, Korea; seonwoo.kim@samsung.com (S.K.); ny.hwang@sbri.co.kr (N.Y.H.)

**Keywords:** personal protective equipment, COVID-19, intubation, intratracheal, laryngoscopes

## Abstract

Various types and levels of personal protective equipment (PPE) are currently available to protect health-care workers against infectious diseases. However, wearing cumbersome PPE may negatively affect their performance in life-saving procedures. This study aimed to evaluate the impact of wearing extensive PPE, including a powered air-purifying respirator with a loose-fitting hood or an N95 filtering facepiece respirator, on the first-pass success (FPS) rate of endotracheal intubation (ETI) in the emergency department (ED). This study was a single-center, observational before-and-after study of 934 adult (≥18 years old) patients who underwent ETI in the academic ED. The study period was divided into a control period (from 20 January 2019, to 30 September 2019, and from 20 January 2018, to 30 September 2018) and an intervention period (from 20 January 2020, to 30 September 2020). Extensive PPE was not donned during the control period (control group, *n* = 687) but was donned during the intervention period (PPE group, *n* = 247). The primary outcome was the FPS rate. We used propensity score matching between the PPE and control groups to reduce potential confounding. Propensity score matching identified 247 cases in the PPE group and 492 cases in the control group. In the matched cohort, no significant difference was found in the FPS rate between the PPE and control groups (83.8% (*n* = 207) vs. 81.9% (*n* = 403); *p* = 0.522). In multivariable analysis, wearing PPE was not associated with the FPS rate (adjusted odds ratio, 0.90; 95% confidence interval, 0.57–1.40; *p* = 0.629) after adjusting for the level of the intubator (junior resident, senior resident, or emergency medicine (EM) specialist). In conclusion, the FPS rate is not significantly affected by wearing extensive PPE in the ED.

## 1. Introduction

Severe acute respiratory syndrome coronavirus 2 (SARS-CoV-2), a novel coronavirus that causes coronavirus disease 2019 (COVID-19), has spread worldwide, leading the World Health Organization to declare a pandemic on 11 March 2020. The global burden of COVID-19 is substantial. As of December 2020, there were more than 80 million laboratory-confirmed cases worldwide, with 1.8 million deaths [1]. As severe morbidities and mortality from COVID-19 are mainly attributed to pneumonia and subsequent acute respiratory distress syndrome, a significant number of patients need invasive airway support. A study of 5700 patients hospitalized for COVID-19 in New York reported that 20.2% of patients required invasive mechanical ventilation [2].

Performing endotracheal intubation (ETI) in patients with COVID-19 carries a high risk for health-care workers (HCWs) [3,4]. SARS-CoV-2 is highly contagious and is known to be transmitted via droplets or even aerosols [5]. ETI can potentially lead to the aerosolization of virally contaminated body fluid [4]. In addition, it is often accompanied by other aerosol-generating procedures, such as airway suctioning, manual ventilation, cardiopulmonary resuscitation, and non-invasive ventilation. In this regard, El-Boghdadly et al. reported that 10.7% of the 1718 HCWs who participated in 5148 ETI episodes for patients with suspected or confirmed COVID-19 were diagnosed with COVID-19, needing self-isolation or requiring hospitalization for new symptoms [6].

The current SARS-CoV-2 pandemic poses significant challenges for front-line HCWs, especially those working in the emergency department (ED) [7]. They often engage in treatment with insufficient information about the patients. Given the current pandemic situation, almost every patient who needs aerosol-generating procedures should be treated as if they are potentially infected by SARS-CoV-2 until proven otherwise. Various types and levels of personal protective equipment (PPE) are currently available to protect HCWs against infectious diseases. However, wearing cumbersome PPE may negatively affect their performance in life-saving procedures. In this context, several studies have investigated the effect of wearing PPE on ETI outcomes but have yielded mixed results [8,9,10,11,12,13,14,15]. However, most of these studies were simulation based, using mannequins or cadavers, and clinical studies conducted in the ED setting are lacking.

In this study, we aimed to evaluate the impact of wearing extensive PPE on the first-pass success (FPS) rate of ETI performed in the ED.

## 2. Materials and Methods

### 2.1. Study Design and Setting

This study was a single-center, retrospective observational before-and-after study conducted in the academic ED, with over 70,000 annual ED visits. This institution provides an accredited four-year emergency medicine (EM) residency program. Approximately 400 ETI procedures are performed annually among adult patients in the ED, and EM physicians are responsible for most ETI procedures in the ED. In addition, the EM specialist on duty supervises the entire procedure for every ETI performed in the ED.

As the new infectious disease caused by the novel coronavirus was reported in China, our emergency medical personnel began to wear extensive PPE when treating suspicious patients. Since the first COVID-19 case in Korea was identified on 20 January 2020, it has been mandatory for all HCWs participating in ETI to wear extensive PPE in the ED. For this study, the intervention period was from 20 January 2020, to 30 September 2020. For comparison, the control period was the same period in each of the previous two years (from 20 January 2019, to 30 September 2019, and from 20 January 2018, to 30 September 2018). The study complied with the Strengthening the Reporting of Observational studies in Epidemiology statement [16]. This study was approved by the Institutional Review Board (IRB), and the need for informed consent was waived because this was a retrospective study and no interventions were performed (IRB number, 2020-09-134-001).

### 2.2. Study Population

All adults (≥18 years old) who underwent ETI performed in the ED during the study period were included in the analysis. The exclusion criteria were as follows: (1) patients who underwent endotracheal tube exchange using a tube exchanger due to tube leakage and (2) patients with ETI who used devices other than the conventional Macintosh-type direct laryngoscope (DL) and C-MAC video laryngoscope (C-MAC VL, Karl Storz Endoskope, Tuttlingen, Germany).

### 2.3. Measures

During the intervention period, extensive PPE was donned, which included a complete bodysuit or at least a waterproof surgical gown, apron, double-gloving, boots, N95 filtering facepiece respirators (N95 respirators), and powered air-purifying respirators (PAPRs) (see Supplementary Materials, Figure S1). In some patients classified as having a very low probability of COVID-19, N95 respirators with face shields or goggles were worn instead of PAPRs. The PAPR used in this study consisted of a loose-fitting hood, a breathing tube, a high efficiency particulate air filter, and a blower. We used two types of PAPRs: (1) 3M Jupiter Powered Air Turbo with a breathing tube (BT-20 L) and a loose-fitting hood (S-433 L-5) (3M, St. Paul, MN, USA) and (2) an AIR WING III PAPR system with a hood kit (OTOS, Seoul, Korea). During the control period, a surgical mask and rubber gloves with/without a disposable chlorinated polyethylene isolation gown were mainly used as a standard practice in our ED.

Direct laryngoscopy was performed using the DL (blade size 3 or 4) and video laryngoscopy using the C-MAC VL. The selection of devices to facilitate ETI was at the discretion of the intubator.

Consecutive ETI cases were registered in our institutional airway registry [17]. ETI data were collected in real time by the monitoring staff to minimize recall and reporting bias. The registry was completed at the end of the procedure by the intubator and the monitoring staff. The EM specialist responsible for the airway registry finally reviewed and confirmed the data. The following data were retrieved from our institutional airway registry and electronic medical records: demographic characteristics of the patients, including age, sex, body mass index (BMI), vital signs, and peripheral blood oxygen saturation measured at the time of the decision to intubate; the presence of difficult laryngoscopy characteristics; methods of ETI; the specialty and level of the intubator; intubating devices; the number of ETI attempts; associated complications; drugs, including sedatives and neuromuscular blocking agents; and glottic view as per the Cormack and Lehane (C-L) grade.

The level of the intubator was categorized as follows: junior resident (first- or second-year resident), senior resident (third- or fourth-year resident), or EM specialist. An ETI attempt was defined as the placement of a laryngoscope blade into the mouth regardless of successful insertion of the endotracheal tube into the trachea. The number of these attempts was strictly counted by the monitoring staff. FPS was defined as successful endotracheal tube placement in the trachea on the first ETI attempt. Multiple attempts were defined as three or more ETI attempts. Anticipated difficult laryngoscopy was determined by the intubator based on the patient’s features, such as the external appearance, including a short neck, facial trauma, a small mandible, or obesity; Mallampati class 3 or higher; airway obstruction, including airway edema or a history of tracheal stenosis; cervical immobilization; and limited mouth opening of <3 cm. Post-intubation hypotension was defined as newly developed hypotension (systolic blood pressure of <90 mmHg) at any time within 30 min after the first ETI attempt that was presumed to be related to ETI. Post-intubation hypoxemia was defined as newly developed hypoxemia (peripheral oxygen saturation of <80%) at any time within 30 min after the first ETI attempt that was presumed to be related to ETI. Unrecognized esophageal intubation was defined as esophageal intubation found after worsening of the patient’s condition. Cardiac arrest was defined as cardiac arrest that occurred within 30 min after the first ETI attempt, regardless of the cause.

The primary outcome measure was the FPS rate. The secondary outcome measures were multiple attempts, glottic view with C-L grade III or IV, and ETI-related complications.

### 2.4. Data Analysis

The ETI cases were classified into a PPE group (ETI performed during the intervention period) and a control group (ETI performed during the control period) for comparison. We used propensity score matching between the PPE group and the control group to reduce potential confounding. A propensity score was calculated using logistic regression analysis with the following covariates: BMI grades of the patients, the presence of difficult laryngoscopy characteristics, ETI methods (crash approach, rapid sequence intubation, sedation only, and no medication), and the ETI device used for the first attempt (DL vs. C-MAC VL). We performed 1:2 matching by the nearest matching method using a caliper of 0.5. A standard mean difference of <0.1 between the PPE group and the control group was used to evaluate matching adequacy.

We presented continuous data as means (standard deviations) and categorical data as numbers (%). The variables before matching were compared using Student’s *t*-test, the chi-square test, or Fisher’s exact test, as appropriate. After propensity score matching, all variables were compared using the generalized estimating equation approach.

The generalized estimating equation approach was also applied to evaluate the relationship between wearing of PPE and the primary and secondary outcomes. The level of the intubator (junior resident, senior resident, or EM specialist) was included as a covariate in this model because it could considerably affect the outcomes. We presented the data as adjusted odds ratios (aORs) and 95% confidence intervals (CIs).

We performed additional subgroup analysis on cases wherein only the C-MAC VL was used. Propensity score matching was performed in the same way as that in the previous process, and the BMI grades of the patients and the presence of difficult laryngoscopy characteristics were used as matching variables. The association between wearing of PPE and outcomes, including the FPS rate, multiple attempts, glottic view with C-L grade III or IV, and ETI-related complications, was also evaluated using a generalized estimating equation approach with adjustment for the level of the intubator and ETI methods.

Statistical significance was set at a *p*-value of <0.05. The data were analyzed using R software (version 3.6.1, R Foundation for Statistical Computing, Vienna, Austria; http://www.R-project.org/; accessed on 31 October 2020).

## 3. Results

### 3.1. Characteristics of Study Subjects

**Figure 1:**
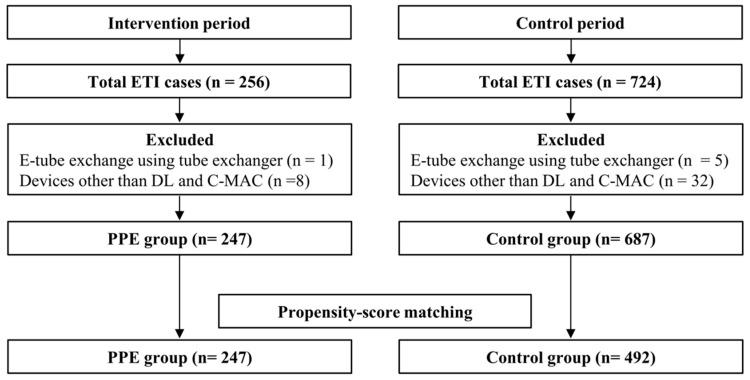
Study flowchart. ETI, endotracheal intubation; E-tube, endotracheal tube; DL, direct laryngoscope; C-MAC, C-MAC video laryngoscope; PPE, personal protective equipment.

A total of 980 ETI procedures were performed during the study period, of which 46 were excluded from the analysis for the reasons mentioned in Figure 1. Of the eligible 934 ETI cases, 247 and 687 were assigned to the PPE and control groups, respectively. Of 71.7% (*n* = 177) of the patients in the PPE group tested for SARS-CoV-2, none tested positive. In the PPE group, a PAPR with a loose-fitting hood was used in the majority of ETI cases (80.2%, *n* = 198). Propensity score matching identified 247 cases in the PPE group and 492 cases in the control group. This method resulted in balanced groups for matching variables, including the BMI grade, anticipated difficult laryngoscopy and intubation, ETI methods, and intubating devices. The baseline characteristics of the patients and ETI procedures are presented in Table 1. The systolic blood pressure was significantly lower both before and after matching in the PPE group (both *p* = 0.001). Both before and after matching, senior residents performed the first attempt most frequently in the PPE group, whereas junior residents performed the first attempt most frequently in the control group (*p* < 0.001).

The crash approach, referred to in Table 1, was used for unconscious, unresponsive patients expected not to be resistant to laryngoscopy and who needed immediate airway security.

### 3.2. Main Results

**Figure 2:**
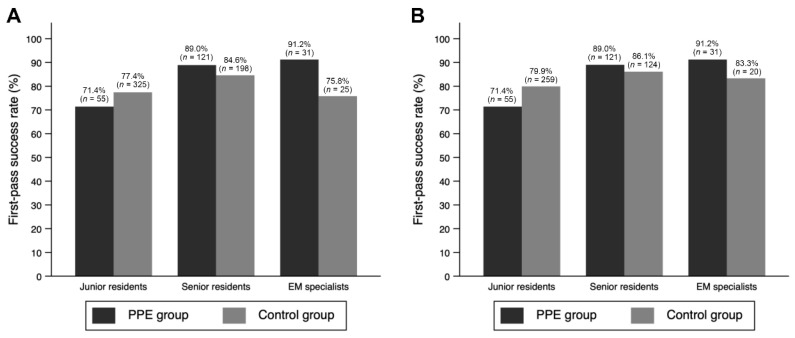
Comparison of the first-pass success rate according to the level of the intubator. (**A**) Before matching. (**B**) After matching. PPE, personal protective equipment; EM, emergency medicine.

The primary and secondary outcomes are presented in Table 2 and Figure 2. In the unmatched cohort, the overall FPS rate was 80.8% (*n* = 755), with 83.8% (*n* = 207) in the PPE group and 79.8% (*n* = 548) in the control group (*p* = 0.168). In the matched cohort, there was no significant difference in the FPS rate between the PPE group and the control group (83.8% (*n* = 207) vs. 81.9% (*n* = 403); *p* = 0.522). Furthermore, there was no significant difference found between the PPE group and the control group in the matched cohort in terms of multiple attempts (4.5% (*n* = 11) vs. 4.9% (*n* = 24); *p* = 0.798), glottic view with C-L grade III or IV on the first attempt (7.7% (*n* = 19) vs. 9.8% (*n* = 48); *p* = 0.354), and overall complication rates (19.0% (*n* = 47) vs. 14.8% (*n* = 73); *p* = 0.146). Post-intubation hypotension was more frequent in the PPE group than in the control group in both unmatched (11.7% (*n* = 29) vs. 3.3% (*n* = 23); *p* < 0.001) and matched cohorts (11.7% (*n* = 29) vs. 3.7% (*n* = 18); *p* < 0.001).

After adjusting for the level of the intubator, wearing of PPE was not found to be associated with the FPS rate (aOR, 0.90; 95% CI, 0.57–1.40; *p* = 0.629), multiple attempts (aOR, 0.90; 95% CI, 0.39–2.07; *p* = 0.795), glottic view (aOR, 0.78; 95% CI, 0.43–1.40; *p* = 0.404), and overall complications (aOR, 1.36; 95% CI, 0.87–2.13; *p* = 0.173) (Table 3). Performance of ETI by a senior resident (aOR, 2.00; 95% CI, 1.27–3.16; *p* = 0.003) was associated with an increased FPS rate compared to that by a junior resident.

### 3.3. Subgroup Analysis of Cases Wherein C-MAC VL Was Used

The results of the subgroup analysis of cases wherein the C-MAC VL was used are presented elsewhere (see Supplementary Materials, Tables S1 and S2). There was no significant difference in the FPS rate between the PPE group and the control group in both the unmatched (88.2% (*n* = 179) vs. 84.0% (*n* = 356); *p* = 0.164) and matched (88.2% (*n* = 179) vs. 84.4% (*n* = 341); *p* = 0.212) cohorts. Other outcomes, including multiple attempts and glottic view with C-L grade III or IV in the first attempt, were also not significantly different between groups in both unmatched and matched cohorts. Overall complications were higher in the PPE group than in the control group in both unmatched (20.2% (*n* = 41) vs. 13.7% (*n* = 58); *p* = 0.037) and matched (20.2% (*n* = 41) vs. 13.9% (*n* = 56); *p* = 0.046) cohorts. Wearing of PPE was not associated with the FPS rate (aOR, 0.99; 95% CI, 0.56–1.74; *p* = 0.960) and other outcomes after adjusting for the level of the intubator and the ETI methods.

## 4. Discussion

In this study, wearing of extensive PPE, including a PAPR with a loose-fitting hood or an N95 respirator, did not affect the FPS rate of ETI performed in the ED. Furthermore, there were no significant differences between other outcomes, including multiple attempts, glottic view, and overall complications. In addition, wearing of PPE was not associated with the FPS rate in the subgroup analysis of cases wherein the C-MAC VL was used. The COVID-19 pandemic has brought ETI techniques into further focus to secure the safety of HCWs in addition to that of patients. In this respect, our findings are highly relevant to the current clinical situation and suggest that wearing extensive PPE may preserve the intubator’s safety without hindering their performance.

Several recent outbreaks, such as that of severe acute respiratory syndrome coronavirus 1, have yielded valuable lessons for HCWs regarding the importance of PPE use [18]. However, there has been continuing concern that wearing PPE will hinder the intubator’s performance, possibly owing to restricted movement, loss of manual dexterity, visual impairment, and limited communication. It is a clinically vital issue as it can seriously affect patient safety. Several studies have evaluated the impact of PPE usage on ETI performance or compared the efficacy of various intubating devices while the intubator was wearing PPE and have yielded conflicting results. In a study that compared the effect of wearing a general surgical gown or antichemical protective gear on ETI performance in the operating room, the ETI time was significantly prolonged due to PPE [8]. In a human cadaver study, wearing level C PPE both impeded the FPS and increased the time to successful ETI, regardless of the device type (DL and McGarth VL) [9]. Conversely, Wong et al. reported that wearing PPE, including a PAPR, did not affect the success rate of ETI in the ED [10]. In a manikin study in the setting of wearing level C PPE, ETI time was longer when using a GlideScope Ranger VL than when using a King Vision VL or DL, which was not significantly different from each other [15]. However, Pentax-AWS with level C PPE showed better performance than the unsuited DL for ETI time [13]. Thus, it is difficult to reach any definite conclusions on the impact of wearing PPE on ETI performance or the superiority of a specific intubating device while wearing PPE. Because the study design and setting, outcomes of interest, competency of participants, devices used, and different levels and types of PPE vary significantly across studies, the study results should be interpreted in these contexts. In our study, wearing of PPE did not affect the outcomes of ETI, and our findings may have several possible explanations. First, the HCWs in our ED were familiar with the PPE used in this study. Our institution experienced extensive nosocomial infections during the 2015 Middle East respiratory syndrome outbreak. Since then, extensive PPE has been used when treating patients suspected of having or confirmed to have high-risk diseases. Second, simulation-based drills have been conducted regularly to maintain the HCWs’ confidence in task performance while wearing PPE, including PAPRs. The role of simulation-based drills in the medical field is well established. These drills provide an opportunity for HCWs to rehearse high-risk procedures such as ETI for COVID-19 patients under real-world conditions [19,20]. They also enable HCWs to better understand their roles and responsibilities, improve communication among team members, and increase teamwork competencies while performing tasks. HCWs should be encouraged to undergo ongoing simulation-based training to prepare themselves appropriately for rapidly changing environments and settling in new processes in the clinical field in a timely manner. Third, it is possible that the PPE used in our study was relatively less bulky, thus impeding the procedure to a lesser degree. In their study, Flaishon et al. attributed their results to the use of cumbersome outfits, including two layers of clothing, thick rubber gloves, and antigas masks [8]. Furthermore, in the study by Taylor et al., participants reported that interference with glottis visualization by hoods may have influenced the results [9]. Conversely, the PPE used in our study was relatively simple compared with that used in the aforementioned studies. In particular, latex gloves seemed to have a minimal effect on the operator’s manual dexterity. Moreover, the face shield attached to the hood was wide and clear, leading to less visual impairment.

The COVID-19 pandemic has led to several changes in airway management. These changes can have a potentially negative impact on the training of less experienced physicians, especially junior residents [21]. For example, recent airway management guidelines for patients with confirmed or suspected COVID-19 advocate ETI being performed by the most experienced physician to increase the FPS rate [22]. However, performing ETI in undifferentiated patients is prevalent in the ED, and if less experienced physicians are excluded from the procedure in these situations, they may miss an opportunity to gain sufficient clinical experience and skills. In this study, the proportion of junior residents performing the first ETI attempt decreased markedly during the intervention period, while that of senior residents and EM specialists increased during the same period. These findings seem to reflect the changes in practice owing to the COVID-19 pandemic. However, the first attempt was performed by residents in more than 80% of all ETI cases. The junior residents still performed more than 30% of the first attempts. Nevertheless, the outcomes related to ETI may not have been significantly affected. Our institution has made various efforts to provide appropriate training to less experienced physicians without compromising the safety of patients and intubators. These include the presence of EM specialists during all ETI procedures, 24 h support from anesthesiologists and otolaryngologists for patients with an anticipated difficult airway, increased use of video laryngoscopes, restriction of the number of ETI attempts to two or fewer by less experienced physicians, and a regular simulation-based drill for airway management while wearing PPE. Unprecedented situations, such as the current COVID-19 pandemic, provide physicians with unique experiences. However, institutional strategies are necessary so that less experienced physicians are not hindered from acquiring clinical expertise and competency [23]. In addition, regular monitoring of their training status would be necessary.

Both before and after matching, post-intubation hypotension was found to be significantly more frequent in the PPE group than in the control group. It is not clear what caused these findings; however, some possible explanations are as follows: First, the implementation of early resuscitation may have been delayed in the PPE group compared with that in the control group. The COVID-19 pandemic has led to a marked increase in the complexity of the ED care process, including triage, bed allocation, and diagnostic and therapeutic approaches. In addition, performing ETI in an isolation room while wearing PPE might further interfere with patient treatment. Therefore, ETI performed in unoptimized patients may be related to the occurrence of post-intubation hypotension. Second, ketamine was used more frequently in the PPE group than in the control group. Ketamine is known to yield hemodynamic stability. However, several studies conducted in the ED suggest that ketamine administration may be related to post-intubation hypotension [24,25]. April et al. evaluated the effects of ketamine and etomidate on the occurrence of peri-intubation hypotension in the context of ETI performed in the ED using a large observational registry [24]. They found that peri-intubation hypotension was more frequent in patients receiving ketamine (18.3% vs. 12.4%). However, further research is needed to clarify this issue.

There are several limitations to consider when interpreting the results of this study. First, this was a single-center study conducted in a tertiary academic ED; thus, the results may not be generalizable to other settings. In particular, since wearing PPE has been frequent in our institution after the 2015 Middle East respiratory syndrome outbreak and training related to wearing PPE has been repeatedly performed, there may be limitations in applying our findings to other settings. Second, because this study was performed retrospectively, the baseline characteristics of the patients and ETI-related factors could not be controlled. There was a significant difference in the levels of the intubator who attempted the first ETI between the two groups. It is well known that an intubator’s experience can affect the success rate of ETI. Therefore, we conducted further analysis of the effect of PPE on the outcomes by adjusting the level of the intubator to minimize potential bias. Third, only the C-MAC VL and DL were used as equipment to facilitate ETI in this study. Therefore, different results may be obtained if other types of equipment are used. In particular, because VLs are of various shapes (e.g., channeled vs. non-channeled, hyper-angulated blade vs. Macintosh-type blade), care should be taken when applying our findings to other settings. Finally, there were many restrictions regarding the environment for the procedure during the intervention period. In particular, only the fewest possible number of necessary HCWs participated in the procedure to minimize the potential risks of infection. Problems such as insufficient assistance or delay in drug preparation may have occurred owing to the reduced workforce, which might have affected the outcomes. However, this aspect was not considered in the analysis, because it was difficult to quantify these problems.

## 5. Conclusions

The FPS rate was not significantly affected by the use of extensive PPE in the ED in this study. The other outcomes, including multiple attempts, glottic view, and overall complications, were not significantly different between the PPE and control groups. Our results would be useful for the planning of precautions for front-line HCWs during ETI. Considering the current pandemic situation, further robust studies are needed to establish the impact of various types of PPE and devices on airway-related outcomes.

## Figures and Tables

**Table 1:** Baseline characteristics of the patients and ETI procedures.

	Before Matching				After Matching *			
	Total (*n* = 934)	PPE Group (*n* = 247)	Control Group (*n* = 687)	*p*-Value	Total (*n* = 739)	PPE Group (*n* = 247)	Control Group (*n* = 492)	*p*-Value
**Patient age (years)**	65.6 (15.3)	66.3 (14.5)	65.4 (15.6)	0.452	65.6 (15.4)	66.3 (14.5)	65.3 (15.8)	0.414
**Patient sex (male)**	578 (61.9)	149 (60.3)	429 (62.4)	0.608	460 (62.2)	149 (60.3)	311 (63.2)	0.463
**Patient BMI (kg/m^2^)**	22.9 (4.0)	22.6 (3.9)	23.1 (4.0)	0.123	22.9 (4.1)	22.6 (3.9)	23.10 (4.2)	0.080
**Patient BMI grade** *				0.422				0.466
Normal (18.5–24.9)	594 (63.6)	159 (64.4)	435 (63.3)		475 (64.3)	159 (64.4)	316 (64.2)	
Under (<18.5)	105 (11.2)	31 (12.6)	74 (10.8)		83 (11.2)	31 (12.6)	52 (10.6)	
Over (25.0–29.9)	195 (20.9)	44 (17.8)	151 (22.0)		146 (19.8)	44 (17.8)	102 (20.7)	
Obesity (≥30.0)	40 (4.3)	13 (5.3)	27 (3.9)		35 (4.7)	13 (5.3)	22 (4.5)	
**Vital signs and SpO_2_** ^†^								
Systolic BP (mmHg)	132.45 (43.0)	123.3 (39.0)	135.90 (43.9)	0.001	131.6 (43.8)	123.3 (39.0)	135.9 (45.5)	0.001
Diastolic BP (mmHg)	73.9 (26.3)	71.9 (25.4)	74.67 (26.6)	0.242	73.7 (26.7)	71.9 (25.4)	74.6 (27.3)	0.257
Heart rate (per minute)	109.2 (26.8)	109.7 (25.2)	109.02 (27.4)	0.789	108.4 (26.9)	109.7 (25.2)	107.7 (27.7)	0.424
Respiratory rate (per minute)	26.1 (9.0)	26.8 (9.8)	25.88 (8.6)	0.254	26.2 (9.0)	26.8 (9.8)	25.8 (8.5)	0.255
SpO_2_ (%)	92.9 (11.0)	93.5 (7.9)	92.68 (11.9)	0.434	93.0 (10.0)	93.5 (7.9)	92.8 (11.0)	0.443
**Difficult laryngoscopy** *	267 (28.6)	73 (29.6)	194 (28.2)	0.756	213 (28.8)	73 (29.6)	140 (28.5)	0.757
**ETI indication ^†^**								
Cardiac arrest	308 (33.0)	74 (30.0)	234 (34.1)	0.418	237 (32.1)	74 (30.0)	163 (33.1)	0.702
Altered mental status	173 (18.5)	44 (17.8)	129 (18.8)		136 (18.4)	44 (17.8)	92 (18.7)	
Respiratory distress	297 (31.8)	79 (32.0)	218 (31.7)		234 (31.7)	79 (32.0)	155 (31.5)	
Shock	77 (8.2)	23 (9.3)	54 (7.9)		64 (8.7)	23 (9.3)	41 (8.3)	
Others	79 (8.5)	27 (10.9)	52 (7.6)		68 (9.2)	27 (10.9)	41 (8.3)	
**Method of ETI** *				0.191				0.237
Crash approach	336 (36.0)	82 (33.2)	254 (37.0)		253 (34.2)	82 (33.2)	171 (34.8)	
RSI	512 (54.8)	135 (54.7)	377 (54.9)		411 (55.6)	135 (54.7)	276 (56.1)	
Sedative only	85 (9.1)	30 (12.1)	55 (8.0)		75 (10.1)	30 (12.1)	45 (9.1)	
No medication	1 (0.1)	0 (0.0)	1 (0.1)		0 (0.0)	0 (0.0)	0 (0.0)	
**First attempt**								
Specialty of the intubator				0.218				0.245
EM	871 (93.3)	235 (95.1)	636 (92.6)		692 (93.6)	235 (95.1)	457 (92.9)	
Non-EM	63 (6.7)	12 (4.9)	51 (7.4)		47 (6.4)	12 (4.9)	35 (7.1)	
Level of the intubator ^‡^				<0.001				<0.001
Junior resident	497 (53.2)	77 (31.2)	420 (61.1)		401 (54.3)	77 (31.2)	324 (65.9)	
Senior resident	370 (39.6)	136 (55.1)	234 (34.1)		280 (37.9)	136 (55.1)	144 (29.3)	
EM specialist	67 (7.2)	34 (13.8)	33 (4.8)		58 (7.8)	34 (13.8)	24 (4.9)	
Device *				<0.001				0.547
DL	307 (32.9)	44 (17.8)	263 (38.3)		133 (18.0)	44 (17.8)	89 (18.1)	
C-MAC VL	627 (67.1)	203 (82.2)	424 (61.7)		606 (82.0)	203 (82.2)	403 (81.9)	
Sedatives				0.082				0.021
Ketamine	144 (15.4)	51 (20.6)	93 (13.5)		115 (15.6)	51 (20.6)	64 (13.0)	
Etomidate	414 (44.3)	100 (40.5)	314 (45.7)		335 (45.3)	100 (40.5)	235 (47.8)	
Midazolam	20 (2.1)	7 (2.8)	13 (1.9)		18 (2.4)	7 (2.8)	11 (2.2)	
Others	26 (2.8)	7 (2.8)	19 (2.8)		24 (3.2)	7 (2.8)	17 (3.5)	
No sedatives	330 (35.3)	82 (33.2)	248 (36.1)		247 (33.4)	82 (33.2)	165 (33.5)	
NMBAs				0.775				0.708
Succinylcholine	230 (24.6)	65 (26.3)	165 (24.0)		189 (25.6)	65 (26.3)	124 (25.2)	
Rocuronium	270 (28.9)	66 (26.7)	204 (29.7)		212 (28.7)	66 (26.7)	146 (29.7)	
Others ^§^	18 (1.9)	4 (1.6)	14 (2.0)		14 (1.9)	4 (1.6)	10 (2.0)	
No NMBAs	416 (44.5)	112 (45.3)	304 (44.3)		324 (43.8)	112 (45.3)	212 (43.1)	

Data are presented as means (standard deviations) or numbers (%). * The propensity score was matched. ^†^ Measured at the time of the decision to intubate. ^‡^ Junior resident refers to first- and second-year residents; senior resident refers to third- and fourth-year residents. ^§^ Vecuronium and cisatracurium were included. PPE, personal protective equipment; BMI, body mass index; SpO_2_, peripheral oxygen saturation; BP, blood pressure; ETI, endotracheal intubation; RSI, rapid sequence intubation; EM, emergency medicine; DL, conventional direct laryngoscope; VL, video laryngoscope: NMBA, neuromuscular blocking agent.

**Table 2:** Primary and secondary outcomes.

	Before Matching				After Matching			
	Total (*n* = 934)	PPE Group (*n* = 247)	Control Group (*n* = 687)	*p*-Value	Total (*n* = 739)	PPE Group (*n* = 247)	Control Group (*n* = 492)	*p*-Value
**Primary outcome**								
First-pass success rate	755 (80.8)	207 (83.8)	548 (79.8)	0.168	610 (82.5)	207 (83.8)	403 (81.9)	0.522
**Secondary outcomes**								
Multiple attempts (≥3)	52 (5.6)	11 (4.5)	41 (6.0)	0.375	35 (4.7)	11 (4.5)	24 (4.9)	0.798
Glottic view								
C-L grade III or IV (%)	98 (10.5)	19 (7.7)	79 (11.5)	0.100	67 (9.1)	19 (7.7)	48 (9.8)	0.354
**Complications**								
Overall complications *	144 (15.4)	47 (19.0)	97 (14.1)	0.068	120 (16.2)	47 (19.0)	73 (14.8)	0.146
EI	34 (3.6)	5 (2.0)	29 (4.2)	0.122	22 (3.0)	5 (2.0)	17 (3.5)	0.286
Unrecognized EI ^†^	1 (0.1)	0 (0.0)	1 (0.1)	0.961	0 (0.0)	0 (0.0)	0 (0.0)	-
Dental injury	15 (1.6)	2 (0.8)	13 (1.9)	0.260	13 (1.8)	2 (0.8)	11 (2.2)	0.182
Post-intubation hypotension	52 (5.6)	29 (11.7)	23 (3.3)	<0.001	47 (6.4)	29 (11.7)	18 (3.7)	<0.001
Post-intubation hypoxemia	31 (3.3)	8 (3.2)	23 (3.3)	0.935	24 (3.2)	8 (3.2)	16 (3.3)	0.992
Vomiting	2 (0.2)	0 (0.0)	2 (0.3)	0.685	2 (0.3)	0 (0.0)	2 (0.4)	0.512 ^‡^
Agitation	6 (0.6)	4 (1.6)	2 (0.3)	0.047	6 (0.8)	4 (1.6)	2 (0.4)	0.109
Cardiac arrest	21 (2.2)	5 (2.0)	16 (2.3)	0.782	20 (2.7)	5 (2.0)	15 (3.0)	0.421
24 h Mortality	5 (0.5)	1 (0.4)	4 (0.6)	0.745	5 (0.7)	1 (0.4)	4 (0.8)	0.531

The data are presented as numbers (%). * Overall complications include esophageal intubation, unrecognized esophageal intubation, dental injury, post-intubation hypotension, post-intubation hypoxemia, vomiting, agitation, cardiac arrest, and death within 24 h after endotracheal intubation. ^†^ Unrecognized EI was defined as EI found after the patient’s condition worsened. ^‡^ Firth correction was used. PPE, personal protective equipment; C-L, Cormack and Lehane; EI, esophageal intubation.

**Table 3:** Relationships between outcomes and the use of PPE among the propensity-score-matched groups.

	OR	95% CI	*p*-Value
**First-pass success**			
**Wearing of PPE**	0.90	0.57–1.40	0.629
**Level of the intubator** *			
Junior resident	Reference		
Senior resident	2.00	1.27–3.16	0.003
EM specialist	2.11	0.86–5.17	0.103
**Multiple attempts**			
**Wearing of PPE**	0.90	0.39–2.07	0.795
**Level of the intubator** *			
Junior resident	Reference		
Senior resident	1.01	0.48–2.15	0.978
EM specialist	1.15	0.28–4.66	0.850
**Glottic view (C-L grade III or IV)**			
**Wearing of PPE**	0.78	0.43–1.40	0.404
**Level of the intubator** *			
Junior resident	Reference		
Senior resident	0.96	0.56–1.65	0.878
EM specialist	0.99	0.35–2.77	0.984
**Overall complications** ^†^			
**Wearing of PPE**	1.36	0.87–2.13	0.173
**Level of the intubator** *			
Junior resident	Reference		
Senior resident	0.89	0.57–1.41	0.894
EM specialist	1.21	0.60–2.45	1.214

* Junior resident refers to first- and second-year residents; senior resident refers to third- and fourth-year residents. ^†^ Overall complications include esophageal intubation, unrecognized esophageal intubation, dental injury, post-intubation hypotension, post-intubation hypoxemia, vomiting, agitation, cardiac arrest, and death within 24 h after endotracheal intubation. OR, odds ratio; CI, confidence interval; PPE, personal protective equipment; C-L, Cormack and Lehane.

## Data Availability

The datasets used and/or analyzed during the current study are available from the corresponding author on reasonable request.

## Supplementary Materials

The following are available online at https://www.mdpi.com/2077-0383/10/5/1060/s1: Figure S1: Physician wearing extensive personal protective equipment. (A) and (B) A complete bodysuit, N95 filtering facepiece respirators, and powered air-purifying respirators with a loose-fitting hood, (C) a waterproof surgical gown and N95 filtering facepiece respirators with a face shield and goggles, Table S1: Outcomes in cases wherein a C-MAC video laryngoscope was used, Table S2: Relationships between outcomes and wearing of PPE in cases wherein a C-MAC video laryngoscope was used.
