# Supplementary material for: Impact of Personal Protective Equipment on the First-Pass Success of Endotracheal Intubation in the ED: A Propensity-Score-Matching Analysis
Source: J Clin Med. 2021 Mar 4;10(5):1060. doi: 10.3390/jcm10051060 (PMC7961519; doi:10.3390/jcm10051060)
Supplement: Supplementary file 1 [file jcm-10-01060-s001.pdf]

Supplementary Materials

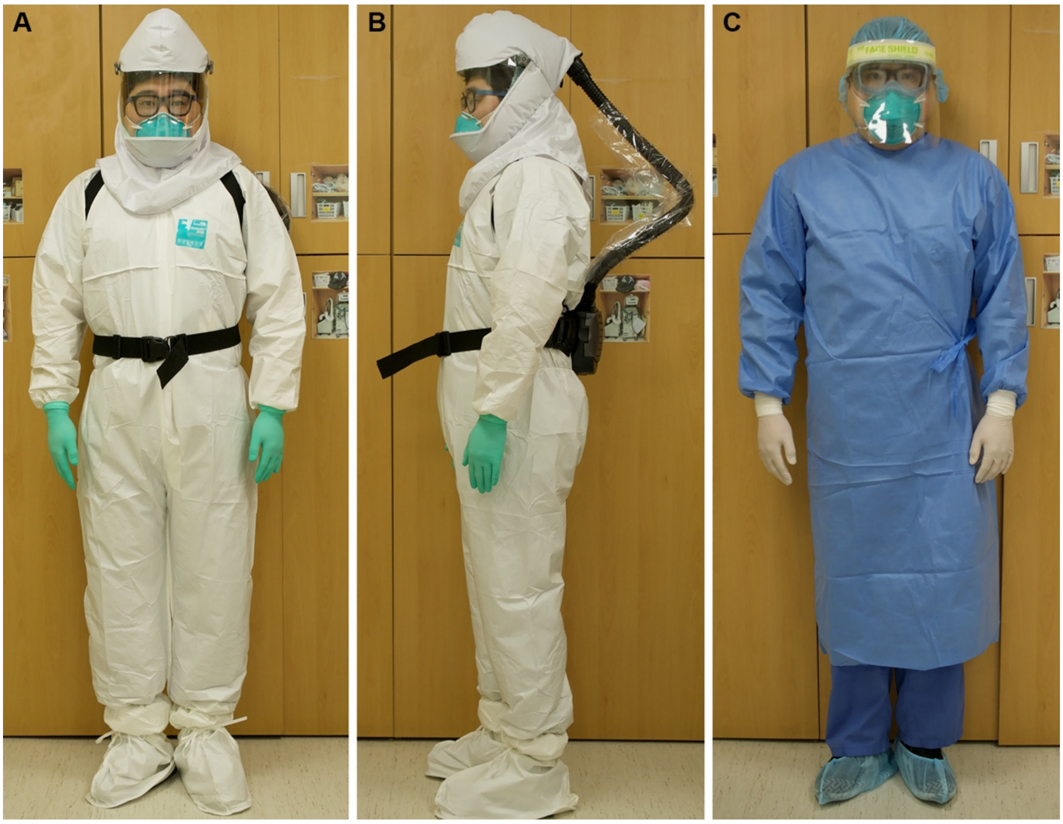

Figure S1. Physician wearing extensive personal protective equipment. A) and B) A complete bodysuit, N95 filtering facepiece respirators, and powered air-purifying respirators with a loose-fitting hood, C) a water-proof surgical gown and N95 filtering facepiece respirators with face shield and goggles.

Table S1. Outcomes in cases wherein a C-MAC video laryngoscope was used.

	Before matching				After matching			
	**Total (*n* = 627)**	**PPE group (*n* = 203)**	**Control group (*n* = 424)**	***p*-value**	**Total (*n* = 607)**	**PPE group (*n* = 203)**	**Control group (*n* = 404)**	***p*-value**
**First-pass success rate**	535 (85.3)	179 (88.2)	356 (84.0)	0.164	520 (85.7)	179 (88.2)	341 (84.4)	0.212
**Multiple attempts (≥ 3)**	22 (3.5)	8 (3.9)	14 (3.3)	0.684	0.722	8 (3.9)	8 (3.9)	0.529
**Glottic view**								
C-L grade III or IV, (%)	40 (6.4)	32 (7.5)	8 (3.9)	0.088	38 (6.3)	8 (3.9)	30 (7.4)	0.099
**Complications**								
Overall complications*	99 (15.8)	41 (20.2)	58 (13.7)	0.037	97 (16.0)	41 (20.2)	56 (13.9)	0.046
EI	13 (2.1)	2 (1.0)	11 (2.6)	0.203	12 (2.0)	2 (1.0)	10 (2.5)	0.230
Dental injury	12 (1.9)	1 (0.5)	11 (2.6)	0.108	12 (2.0)	1 (0.5)	11 (2.7)	0.098
Post-intubation hypotension	42 (6.7)	27 (13.3)	42 (6.7)	<0.001	42 (6.9)	27 (13.3)	15 (3.7)	<0.001
Post-intubation hypoxemia	23 (3.7)	7 (3.4)	16 (3.8)	0.839	23 (3.8)	7 (3.4)	16 (4.0)	
Agitation	6 (1.0)	4 (2.0)	2 (0.5)	0.097	6 (1.0)	4 (2.0)	2 (0.5)	0.109
Cardiac arrest	16 (2.6)	5 (2.5)	11 (2.6)	0.922	14 (2.3)	5 (2.5)	9 (2.2)	0.855
24-h mortality	5 (0.8)	1 (0.5)	4 (0.9)	0.560	5 (0.8)	1 (0.5)	4 (1.0)	0.531

The data are presented as numbers (%).

*Overall complications include esophageal intubation, unrecognized esophageal intubation, dental injury, post-intubation hypotension, post-intubation hypoxemia, vomiting, agitation, cardiac arrest, and death within 24 h after endotracheal intubation.

PPE, personal protective equipment; C-L, Cormack and Lehane; EI, esophageal intubation.

Table S2. Relationships between outcomes and wearing PPE in cases wherein a C-MAC video laryngoscope was used.

	**OR**	**95% CI**	***p*-value**
**First-pass success**			
Wearing of PPE	0.99	0.56-1.74	0.960
**Multiple attempts**			
Wearing of PPE	1.21	0.45-3.26	0.707
**Glottic view (C-L grade III or IV)**			
Wearing of PPE	0.55	0.23-1.34	0.187
**Overall complications***			
Wearing of PPE	1.51	0.95-2.41	0.083

The propensity score-matched cohort was analyzed.

The association between wearing PPE and the outcomes was evaluated using the generalized estimating equation approach with adjustment for the level of the intubator and the ETI methods.

*Overall complications include esophageal intubation, unrecognized esophageal intubation, dental injury, post-intubation hypotension, post-intubation hypoxemia, vomiting, agitation, cardiac arrest, and death within 24 h after endotracheal intubation.

OR, odds ratio; CI, confidence interval; PPE, personal protective equipment; C-L, Cormack and Lehane.
